# New Medical Society

**Published:** 1874-05-15

**Authors:** E. E. Waggoner

**Affiliations:** Sec’y.; Shelbyville, Ill.


NEW MEDICAL SOCIETY. — According to previous notice, quite a respectable number of physicians from the counties of Montgomery, Christian, Shelby, Fayette and Marion, met in the city of Pana, on the 28th ultimo, and organized "THE DISTRICT MEDICAL SOCIETY OF CENTRAL ILLINOIS," by adopting a Constitution and By-Laws, and electing the following officers:

*President*—H. H. Hood, of Taylorville.

*Vice-Presidents*—I. W. Fink, of Hillsboro, and H. H. Deming, of Pana.

*Treasurer*—L. B. Slater, of Taylorville.

*Secretary*—E. E. Waggoner, of Shelbyville.

C. V. Rockwell, of Taylorville, and J. D. Bennett, of Assumption, were appointed delegates to represent this Society at the next meeting of the State Medical Society.

C. V. Rockwell, of Taylorville, and R. E. Beach, of Patoka, were appointed delegates to the next meeting of the National Medical Association.

After the organization was completed, the remainder of the day was spent in an irregular, free-and-easy interchange of thought upon various topics of interest to the profession.

Having a membership of *thirty-one*, and an unmistakable promise of a bright and glorious future, "The District Medical Society of Central Illinois" adjourned at nine o'clock P. M., to meet again on the second Tuesday in July next.

Yours truly,

E. E. WAGGONER, Sec'y.

Shelbyville, Ill., May 5, 1874.

